# Editorial: *In vitro* cellular technologies for advancing research in anamniote vertebrate immunology

**DOI:** 10.3389/fimmu.2023.1277466

**Published:** 2023-10-02

**Authors:** Nguyen T. K. Vo, Tamiru N. Alkie, Alberto Cuesta, Eva-Stina I. Edholm

**Affiliations:** ^1^ Department of Health Studies, Faculty of Human and Social Sciences, Wilfrid Laurier University, Brantford, ON, Canada; ^2^ Department of Health Sciences, Faculty of Science, Wilfrid Laurier University, Waterloo, ON, Canada; ^3^ Immunobiology for Aquaculture Group, Department of Cell Biology and Histology, Faculty of Biology, University of Murcia, Murcia, Spain; ^4^ Norwegian College of Fishery Science, Faculty of Biosciences, Fisheries & Economics, University of Tromsø—The Arctic University of Norway, Tromsø, Norway

**Keywords:** editorial, fish, amphibian, *in vitro* technique, cell and tissue culture, inflammation, epigenetics, immunotoxicology

*In vitro* cellular technologies nowadays are indispensable tools, adding tremendous values in the advancement of comparative immunology research. These technologies encompass isolation of primary cells from embryos and mature species, applications of permanent/continuous cell lines to study innate and adaptive immune functions/responses, and the development of novel imaging methods to visualize intracellular processes in immune cells. For many decades, cellular technologies have been actively sought and used to respond to specific scientific research needs, ultimately in the pursuit of making discoveries as well as translating knowledge into practical applications. This Research Topic collection in the Frontiers in Immunology journal focuses on teleost fish and amphibian immune systems. Through knowledge dissemination in both original research and critical literature review approaches, this Research Topic includes five articles from researchers around the world who reported advances in *in vitro* cellular technologies that provided new insights into our understanding of anamniote vertebrate immunology.

The first article was a contribution from Tao et al. from China and Portugal. Their research focused on the regulatory role of a microRNA named miR-142a-3p in inflammatory responses in grass carp (*Ctenopharyngodon idella*) infected with *Aeromonas hydrophila* (a Gram-negative bacterium). *A. hydrophila* causes serious inflammatory conditions in infected grass carp; therefore, unravelling the inflammatory processes involved is important to understand the disease progression. As part of the epigenetic programming, microRNAs bind to the target regions of specific genes, leading to the activation or suppression of a series of immunological responses. The authors identified the targets of miR-142a-3p to be *tnfaip2* and *glut3* genes. They found that *A. hydrophila* infection caused increased miR-142a-3p expressions in infected fish tissues. Using the permanent grass carp kidney CIK cell line, the authors demonstrated that miR-142a-3p promoted cell death. As the kidney is a primary hematopoietic tissue in fish, the outcome of cellular death in the kidney could result in severe and generalized pathophysiological alterations. The authors also used primary cultures of Kupffer cells (tissue resident macrophages) to further explore the roles of miR-142a-3p. They revealed that miR-142a-3p down-regulated pro-inflammatory responses and enhanced anti-inflammatory responses in Kupffer cells. Overall, their work provides new insights into the involvement of microRNAs in general and miR-142a-3p in particular in inflammatory responses during bacterial infections in grass carp. To this end, grass carp primary cultures and cell lines are valuable *in vitro* tools for generating accurate and rapid immunological data that align well with *in vivo* findings.

The second article was originated from Soliman et al. from Canada. Their research was focused on mapping the profile and kinetics of leukocyte biology and responses during the acute phase of cutaneous inflammatory conditions in goldfish (*Carassius auratus*). Isolating leukocytes from connective tissue-dense organs such as the skin of fish and amphibians is difficult and cumbersome; however, the authors successfully innovated a leukocyte isolation method specific for fish skin – a major accomplishment to furthering their research. The study revealed that neutrophils and monocytes/macrophages constituted the majority of the inflammatory leukocytes in goldfish skin. This correlated well with the pro-inflammatory gene expression profile. Furthermore, the authors performed a flow cytometry-based imaging technique to visualize the profile and kinetics of reactive oxygen species and nitric oxide production in isolated skin leukocytes. Overall, their research allows us to have a more defined picture of the phagocyte profile that contributes to the induction and resolution of cutaneous inflammation conditions in fish.

The third article contributed by Wu et al. from China and the United States of America explored plasma cell-like population in the head kidney of Nile tilapia (*Oreochromis niloticus*). They sorted IgM^+^ B cells from a whole head kidney leukocyte preparation using flow cytometry and further characterized the cell phenotype with confocal and electron microscopy and cytological staining. Furthermore, they analyzed the spectrum of gene and protein expressions and functional assays and confirmed that these IgM^+^ bearing B cells in the head kidney of tilapia resembled plasma cells. Their research enriches our understanding of the role of fish B lymphocytes in the adaptive immune system.

The fourth article was a comprehensive review paper by Segner et al. from Switzerland, Spain, and China. They discussed in detail the current state of *in vitro* assays using primary and permanent/continuous cell lines to assess immunotoxicological effects of various chemicals in teleost fishes. The authors also identified the limitations of *in vitro* cell-based assays in ecotoxicological risk assessments of fish immune functions. While many efforts have been made to advance the field of *in vitro* immunotoxicology for teleost fishes, the authors emphasized that more collaborative works must be done to apply the predictive potential of results generated in *in vitro* assays to have as an impactful contribution as the *in vivo* systems in decision making.

The fifth article contribution was from Tian et al. from the United States of America. Their research identified microRNAs and non-coding regulatory elements from the whole transcriptome of an amphibian-infecting ranavirus. The authors further showed using *in vitro* frog kidney A6 cells that some of these viral miRNAs targeted and silenced important genes that were part of the interferon-mediated antiviral immune pathways. Collectively, their work demonstrated the existence of an epigenetic co-evolution mechanism at play between a frog virus and its host’s innate antiviral immune responses.

In summary, this Research Topic collection highlights the innovation of *in vitro* cellular technologies in various areas in anamniote vertebrate immunology. The five article contributions clearly demonstrate that more than ever these technologies are technical catalysts that are indispensable in research to allow us to shed new lights on immunological functions in teleost fishes and amphibians.

## Author contributions

NV: Writing – original draft, Writing – review & editing. TA: Writing – review & editing. AC: Writing – review & editing. E-SE: Writing – review & editing.

